# Fatal elective DDD-pacemaker implantation

**DOI:** 10.1007/s12471-017-1048-2

**Published:** 2017-10-30

**Authors:** B. Klop, L. J. P. M. van Woerkens, M. Bijl

**Affiliations:** Department of Cardiology, Albert Schweitzer Hospital, Dordrecht, The Netherlands

A 76-year-old patient with a medical history of hypertension, type 2 diabetes mellitus and moderate valvular aortic stenosis underwent cephalic DDD pacemaker implantation because of fatigue and a 3^rd^ degree atrioventricular block with a ventricular escape rhythm of 45 beats per minute. After implantation, he complained about stabbing chest pains. A chest radiograph did not reveal any signs of a pneumothorax and echocardiography showed normal left ventricular function and no pericardial effusion. Initial electrocardiography showed normal atrioventricular pacing with a premature atrial complex after every two beats and an expected left bundle branch block pattern. Analgesics were started. As complaints persisted, repeat echocardiography revealed deterioration of left ventricular function. We also repeated electrocardiography. Fig. 1 shows the repeat electrocardiogram.

**Fig. 1:**
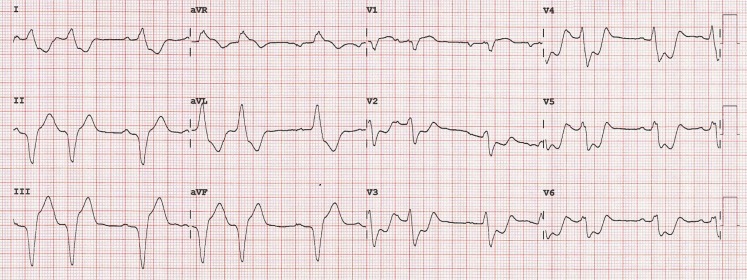
Image of the repeat electrocardiogram

Question: What is the origin of the persisting chest discomfort?

## Answer

You will find the answer elsewhere in this issue.

